# Pilot Data on the Feasibility And Clinical Outcomes of a Nomegestrol Acetate Oral Contraceptive Pill in Women With Premenstrual Dysphoric Disorder

**DOI:** 10.3389/fendo.2021.704488

**Published:** 2021-09-24

**Authors:** Emily Robertson, Caroline Thew, Natalie Thomas, Leila Karimi, Jayashri Kulkarni

**Affiliations:** The Monash Alfred Psychiatry Research Centre, The Alfred and Monash University Central Clinical School, Monash University, Melbourne, VIC, Australia

**Keywords:** nomegestrol acetate/17-beta estradiol, mood, premenstrual dysphoric disorder, depression, contraception

## Abstract

**Background:**

Up to 80% of reproductive-aged women experience premenstrual symptoms. Premenstrual Dysphoric Disorder (PMDD) is a severe form, affecting 2-5% of women. Combined oral contraceptive pills (COCPs) are used in the treatment of PMDD. Clinical practice suggests that a newer COCP containing nomegestrol acetate (2.5mg) and 17-beta estradiol (1.5mg), may be a suitable treatment for mood symptoms in PMDD.

**Materials and Methods:**

This was a clinical follow-up feasibility study of women who had attended the Monash Alfred Psychiatry research centre, Women’s Mental Health Clinic, with a diagnosis of PMDD. 67% of the sample also had concurrent cPTSD, 29% co-morbid anxiety, and 20% depression. They were recommended treatment with nomegestrol acetate/17-beta estradiol. Eligible women were contacted by telephone to answer a questionnaire to assess women’s subjective response to nomegestrol acetate/17-beta estradiol, acceptability and the Depression, Anxiety and Stress Scale-21 (DASS-21) after being recommended nomegestrol acetate/17-beta estradiol. The paired-sample t-test was used to determine if there were any statistically significant differences in the DASS-21 scores over the study observation period (before and after taking nomegestrol acetate/17-beta estradiol).

**Results:**

35 (74.5%) women reported a subjective positive mood response to nomegestrol acetate/17-beta estradiol, 31 (63.3%) adhered to the medication, and only 10 (20.4%) women reported side effects as the main reason for discontinuing nomegestrol acetate/17-beta estradiol. There were statistically significant reductions (*p<*0.05) in the overall DASS-21 scores from before women commenced nomegestrol acetate/17-beta estradiol and after commencement of treatment.

**Conclusions:**

This preliminary study supports the acceptability and effectiveness of nomegestrol acetate/17-beta estradiol as a treatment for mood symptoms in PMDD. Further research, particularly a randomized controlled trial, is required to elucidate the effect of nomegestrol acetate/17-beta estradiol treatment on mood in PMDD.

## Introduction

The prevalence of premenstrual symptoms in women of reproductive age is reported to be up to 80% (1). At the severe end of the spectrum is premenstrual dysphoric disorder (PMDD), affecting 2-5% of women (2). The mood symptoms associated with PMDD cause clinically significant distress, interfering with the ability to work, quality of home life and interpersonal relationships and contributing to a high degree of morbidity in this population (3).

The gonadal hormones (estrogen, progesterone and testosterone) have been shown to influence neurochemistry, brain function and the activity of neurotransmitters gamma-aminobutyric acid (GABA), serotonin and dopamine *via* genomic and non-genomic activity at their respective receptors (4). A recent, comprehensive systematic literature review has summarized the neuroimaging findings demonstrating structural, neurochemical and functional markers of PMDD. The functional brain alterations reported in PMDD involve activation of the amygdala, insular cortex and involvement of the cerebellum, and prefrontal areas. Both hyperactivity of the amygdala and hypoactivity of the PFC are associated with PMDD symptoms severity during the late luteal phase of the menstrual cycle (5).

There is also evidence that estrogen is neuroprotective, in many different brain regions, including the brain stem, with concomitant improvements in mood symptoms (6).

Progesterone, however, can contribute to worsening of mood symptoms in susceptible women, evidenced by worsening of mood symptoms in women who use progesterone-only forms of contraception such as the progesterone-only pill and the levonorgestrel intrauterine device (7). Depressive symptoms in women with PMDD are correlated with these hormonal fluctuations; that is, depression worsens in the premenstrual phase, when estrogen is at its lowest and symptoms improve with the onset of menses (2), suggesting that women with PMDD have an abnormal sensitivity to the hormonal fluctuations that occur across the menstrual cycle. In particular, it is believed that women with PMDD have an abnormal GABA response to changes in allopregnanolone levels (a metabolite of progesterone) across the menstrual cycle, contributing to negative mood symptoms (8).

Management options for PMDD include non-pharmacological treatments, hormonal treatments such as the combined oral contraceptive pill (COCP), estrogen patches and micronized progesterone, as well as antidepressants either intermittently or continuously, or a combination of treatments. If these pharmacological treatments are unsuccessful, gonadotropin-releasing hormone (GnRH) analogues and surgery (hysterectomy and bilateral oophorectomy) with menopausal hormone therapy may be required (4).

Given that fluctuations in hormones clearly contribute to PMDD symptom on and off-set, management with hormonal treatment such as the COCP to stabilize the gonadal hormones is an appropriate option. COCPs have mixed evidence in the management of mood symptoms. While studies suggest some COCPs worsen mood symptoms (7, 9), others suggest there may be no impact on mood in most women (10–13), and others report improvement in mood symptoms with hormonal treatment (14–16). The discrepancies that exist between studies to date could be due to the population studied, the COCP preparation, including the type of progesterone and if the COCP is taken continuously (skipping the inactive pills and taking the active tablets only continuously) or intermittently.

Studies of newer generation COCPs containing antimineralocorticoid and antiandrogenic progestins such as drospirenone are promising (4, 14, 17). COCPs containing antiandrogenic progestins are believed to have less adverse mood effects as they block androgenic hormone properties associated with irritability, a key feature of PMDD (18, 19). An initial study by Freeman et al (20) showed that women with PMDD treated with drospirenone (3mg) and ethinylestradiol (30mcg) (Yasmin), had a reduction in PMDD symptoms compared to placebo. Similar findings were identified by Pearlstein et al (21) and Yonkers et al (22). A subsequent Cochrane review article by Lopez, Kaptein and Helmerhorst (23) concluded that drospirenone containing COCPs may help in the management of PMDD, but that there is also a large placebo effect.

Nomegestrol acetate (2.5mg) and 17-beta estradiol (1.5mg) (Zoely, Merck Sharp & Dohme) is a newer COCP that has been successful in clinical practice for the off-label treatment of mood symptoms in PMDD. Nomegestrol acetate/17-beta estradiol is a monophasic preparation with an extended regimen of 24 active pills followed by four placebo pills (24). It contains a synthetic estrogen (17-beta estradiol) that is structurally identical to endogenous estrogen, whereas most other COCPs contain ethinylestradiol. 17-beta estradiol can cross the blood brain barrier, interact with serotonin receptors and regulate cerebral blood flow to the amygdala and dorsolateral prefrontal cortex, and many other areas of the brain, including important mood-control brainstem centers, all involved in depression (25).

Nomegestrol is structurally similar to progesterone and has strong antigonadotrophic and moderate antiandrogenic activity and no effect on estrogenic, glucocorticoid or mineralocorticoid activity (26). A pooled analysis by Witjes et al (27)of the randomized, open-label, multicenter studies by Mansour et al (28) and Westhoff et al (29) compared the effect of nomegestrol acetate/17-beta estradiol versus drospirenone/ethinylestradiol on premenstrual and menstrual symptoms in healthy women, using the Moos Menstrual Distress Questionnaire Form C (MDQ-C). Women taking nomegestrol acetate/17-beta estradiol experienced a significant improvement in the pain, water retention, negative affect, impaired concentration and behavior change domain scores in the menstrual phase compared with drospirenone/ethinylestradiol users. Arousal scores worsened with nomegestrol acetate/17-beta estradiol but not with drospirenone/ethinylestradiol. The authors concluded that nomegestrol acetate/17-beta estradiol is significantly associated with improvements in many of the MDQ-C domain scores compared with drospirenone/ethinylestradiol (27).

Currently, drospirenone containing COCPs are the recommended COCPs for the treatment of PMDD (4). To date, there have not yet been any studies investigating the impact of nomegestrol acetate/17-beta estradiol treatment on mood in women with PMDD.

This study was undertaken to determine the acceptability and the subjective opinion of nomegestrol acetate/17-beta estradiol treatment on mood in women with PMDD and to determine the changes in the DASS-21 scores before and after nomegestrol acetate/17-beta estradiol treatment in women with PMDD.

## Materials and Methods

### Study Design

This study was a single centre clinical follow-up study that included a questionnaire formulated by the authors (ER and JK) to assess the acceptability and women’s subjective response to nomegestrol acetate/17-beta estradiol and the clinical outcomes using self-report questionnaire the DASS-21. Project 285/17 WMHC Database was approved by the Alfred Hospital Ethics Committee on July 3^rd^, 2020.

### Study Participants

Study participants were identified from the Monash Alfred Psychiatry research centre (MAPrc) Women’s Mental Health Clinic (WMHC) database. The WMHC provides second opinion consults by expert psychiatrists and psychologists for women with a range of psychiatric disorders. The clinic carefully considers the impact of hormonal changes in the management of mental illness. Women who had attended the WMHC at MAPrc from January 2018 to June 2020, had a clinic diagnosis of PMDD, and had been recommended nomegestrol acetate/17-beta estradiol were included. The diagnosis of PMDD was based on a modified clinical assessment using the central 4 items of the Caroline Premenstrual Assessment Scoring System (C-PASS) (30) - the severity of depressive symptoms, duration of symptoms in the premenstrual phase, relative symptom change from pre to post menstrual phase and absolute clearance of symptoms in the post menstrual phase. Hence a semi-structured C-PASS was used to diagnose PMDD.

Only women who had previously completed the WMHC database Participant Consent Form were eligible for inclusion. Women were excluded if they had never taken the recommenced treatment of nomegestrol acetate/17-beta estradiol or taken nomegestrol acetate/17-beta estradiol for less than one month. If women had previous self-harming behavior or a suicide attempt soon after taking nomegestrol acetate/17-beta estradiol they were excluded for safety reasons. Confidentiality was ensured and verbal consent gained from the participants at the start of the follow-up phone call.

Data (demographics, early life trauma, and DASS-21) was collected as part of the participants’ clinical appointment at the WMHC. Menopausal status (reproductive or perimenopausal) was determined by the treating clinician at the clinic appointment based on a comprehensive assessment, including questions about the menstrual status and menopausal symptoms. Follow-up phone calls were made to all eligible participants (those patients with PMDD who have been recommended nomegestrol acetate/17-beta estradiol), and were approximately 20 minutes in length. During this follow-up phone call, the participants completed the DASS-21 scale and were asked questions about their use of nomegestrol acetate/17-beta estradiol, if they take it continuously, side effects of nomegestrol acetate/17-beta estradiol, their subjective opinion of the effect of nomegestrol acetate/17-beta estradiol on their mood and other treatments that they were previously or currently taking.

### The Depression, Anxiety, and Stress Scale-21

The DASS-21 is a validated and reliable self-report questionnaire to measure the negative emotional states of depression, anxiety and stress (31, 32). In a clinical population, the DASS-21 has been shown to accurately represent clinical status and changes after treatment (33).The scale has a total of 21 self-report items with responses rated on a 4-point Likert scale ranging from 0 (never) to 3 (almost always). Higher scores suggest a higher level of depression, anxiety and stress. Given the small sample size of this study, only the overall score of DASS-21 was considered for data analysis purposes.

### Statistical Analysis

The analysis was performed using the IBM statistics program, SPSS (version 26; IBM Corp., Armonk, NY). To determine if continuous data (DASS-21 pre-post overall scores) were normally distributed, Shapiro-Wilk test for normality was used and histograms were generated. The skewness and kurtosis were within the acceptable range and the overall normality results showed no major violation of normality (*p*>0.01). To determine if there were any statistically significant differences in DASS-21 scores between the first visit and the follow-up call, the paired-t test was used. Given the sample size was relatively small and collected from a more heterogeneous patient population, and to reduce any potential bias in the data, we used bootstrapping technique to reduce its impact by generating a bias-corrected (BCa) confidence interval. Thus, using bootstrapping, 1000 random samples were generated to determine the BCa confidence interval to further support the robustness of the results.

Additional sensitivity analyses were carried out by running the same analysis using the non-parametric Wilcoxon signed-rank to check for the robustness of the assumptions. Also, sensitivity analyses were conducted one by one to check if the findings would change with specific subgroups of participants and/or for the key baseline and medical cofounding variables; The variables included age, history of posttraumatic stress disorder (PTSD), smoking, alcohol use, concurrent anxiety, depression, and complex post traumatic stress disorder (cPTSD), taking a selective serotonin reuptake inhibitor (SSRI), antidepressant, antipsychotic, benzodiazepine, anticonvulsant, current use of estrogen patch, and menopausal status (reproductive or peri-menopausal).

## Results

The mean age of the 49 women included in this study was 36 years (sd=8.07). The number of clinic visits during the study period ranged from one to four with a median of two clinic visits (**Table 1**).

**Table 1:** Participant characteristics.

Characteristic	n (%)*
*Age (mean, sd)*	36.1 (8.07)
*Location*	
** **Metropolitan	35 (71.4%)
** **Regional/rural	14 (28.6%)
*Employment*	
** **Employed	34 (70.8%)
** **Unemployed	9 (18.8%)
** **Student	5 (10.4%)
*Education*	
** **Up to 12	6 (12.5%)
** **Vocational training	12 (25.0%)
** **Bachelor's degree	20 (41.7%)
** **Post-graduate degree	10 (20.8%)
*Living situation*	
** **With others	36 (87.8%)
** **Alone	5 (12.2%)
*Relationship status*	
** **Partnered	31 (64.6%)
** **Single	17 (35.4%)
*Smoking*	
** **No	39 (84.8%)
** **Yes	7 (15.2%)
*Alcohol*	
** **No	21 (44.7%)
** **Yes	26 (55.3%)
*Recreational drugs*	
** **No	41 (89.1%)
** **Yes	5 (10.9%)
*Menopausal status*	
** **Reproductive	40 (81.6%)
** **Peri-menopausal	9 (18.4%)
*Menstrual cycles*	
** **Regular	29 (70.7%)
** **Irregular	12 (29.3%)
*History of trauma*	
** **No	5 (10.2%)
** **Yes	44 (89.8%)
*Number of clinic visits since 2018-2020 (median, range)*	2 (1-4)

*n varies from 41-49 due to some missing data for demographic characteristics.

The clinical characteristics of the participants are summarized in **Table 2**. The majority of women were at the reproductive stage (81.6%), with the remaining women being perimenopausal. The majority of women (89.8%) had a history of trauma, including physical, emotional, sexual, peer and neglect. All women had a diagnosis of PMDD, with 33 (67.3%) women having a concurrent diagnosis of complex posttraumatic stress disorder (cPTSD), 28.6% of women had co-morbid anxiety and 20.4% had co-morbid depression, 18 (46.2%) women were taking an SSRI, 10 (25.6%) women were taking another antidepressant, 8 (20.0%) were taking an antipsychotic, 6 (15.4%) were taking a benzodiazepine and 13 (32.5%) were taking an anticonvulsant.

**Table 2:** Participant clinical characteristics.

Characteristic	n	n (%)*
***Comorbidity of cPTSD*** ***No*** ***Yes***	**49**	16 (32.7%) 33 (67.3%)
***Depression*** ***No*** ***Yes***	49	39 (79.6%) 10 (20.4%)
**Anxiety** ** No** **Yes**	49	35 (71.4%) 14 (28.6%)
**Current use of SSRI** **No** **Yes**	39	21 (53.8%) 18 (46.2%)
**Other Antidepressants** **No** **Yes**	39	29 (74.4%) 10 (25.6%)
**Antipsychotics** **No** **Yes**	40	32 (80.0%) 8(20.0%)
**Benzodiazepine** **No** **Yes**	39	33 (84.6%) 6 (15.4%)
**Anticonvulsant** **No** **Yes**	40	27 (67.5%) 13 (32.5%)

*n varies from 41-49 due to some missing data for demographic characteristics.

The current and past hormonal treatment and the patients’ opinion on its effectiveness on mood were reported in **Table 3**. Around 85.7% of women had previously used a different COCP, including ethinylestradiol and levonorgestrel or drospirenone combinations. Use of a progesterone-only treatment in the past was reported by 19 (38.8%) women. 38.1% of the women who had previously taken a different COCP reported a negative mood response; while 68.4% of women taking a progesterone only treatment reported a negative mood response (**Table 3**).

**Table 3:** Other hormonal treatment and subjective opinions on their mood response compared to Nomegestrol acetate/17-beta estradiol.

Treatment	
***Past combined oral contraceptive pill (COCP)*** **Never** **Yes to any** (Ethinylestradiol/levonorgestrel, Ethinylestradiol/norethisterone, Ethinylestradiol/drospirenone, Ethinylestradiol/cyproterone)	n=49 7 (14.3%) 42 (85.7%)
***Progesterone only treatment*** ^b^ **Previous** **Current**	19 (38.8%) 1 (2.0%)
***Estrogen patch*** **Previous** **Current**	14 (28.6%) 13 (26.5%)
***Subjective mood response to previous COCP*** **^a^** **Positive** **Neutral** **Negative** **Missing info**	n=42 6 (14.3%) 14 (33.3%) 16 (38.1%) 6 (14.3%)
***Subjective mood response to previous progesterone only treatment^c^*** **Positive** **Neutral** **Negative** **Missing info**	n=19 0 (0.0%) 4 (21.1%) 13 (68.4%) 2 (10.5%)
***Subjective opinion of Nomegestrol acetate/17-beta estradiol on mood*** **Positive** **Neutral** **Negative**	n=47 35 (74.5) 6 (12.8) 6 (12.8)

a n=42.

b Progesterone only treatment includes minipill, Mirena, Implanon, prometrium.

c n=19.

All women had taken nomegestrol acetate/17-beta estradiol at some point after their first clinical appointment at WMHC. High adherence was reported, with 31 (63.3%) women still currently taking nomegestrol acetate/17-beta estradiol. Nearly half (46.9%) of the women had taken nomegestrol acetate/17-beta estradiol for more than one year with most women (71.4%) taking it continuously. Out of 18 (36.7%) women who had discontinued nomegestrol acetate/17-beta estradiol, side effects were the most commonly provided reason (55.6%), 4 (22.2%) women reported that nomegestrol acetate/17-beta estradiol did not help with their mood, 5.6% of women stated medical reasons for stopping treatment and 5.6% of women were attempting to conceive or pregnant.The most common side effects reported were breakthrough bleeding (40.8%) and decreased libido (24.5%). Thirty five (74.5%) women reported a subjective positive effect of nomegestrol acetate/17-beta estradiol on their mood, 6 (12.8%) reported a neutral effect and 6 (12.8%) reported a negative effect (**Table 4**).

To evaluate the secondary outcome of the study; the clinical effectiveness of nomegestrol acetate/17-beta estradiol, a paired-sample t-test was used to assess the mean differences of the DASS-21 overall scores. The mean, SD and SE of DASS-21 scores from the clinic visit and phone call are displayed in**.** **Table 5**. After checking for normality assumption and outliers the paired-sample t-test was used to assess mean differences. On average, patients reported improved mood change from the clinic visit (M= 27.27, SE=2.00), to follow-up phone call (i.e. before and after taking nomegestrol acetate/17-beta estradiol) (M = 17.03, SE = 1.65) using the self-reported overall score of DASS-21. The difference, 10.23, BCa 95% CI [646, 14.26], was significant t(29) = 4.72, p = 0.001 and represented a large effect size, d =0.86. The post-hoc power anlaysis showed achieved power of 0.99 for this study (**Table 5**). Additional sensitivity analyses were carried out to check the robustness of the findings. A similar significant result found when a non-parametric Wilcoxon Signed Rank test was used to compare the median instead of mean for DASS-21 overall scores (*p*<0.05). In addition, series of sensitivity analyses were conducted on specific subgroups of participants for some key baseline characteristics or potential cofounders one by one. The sensitivity analyses showed similar significant mean differences for groups with no alcohol use, no smoking, no concurrent depression, anxiety or cPTSD, no current use of an estrogen patch, no use of an SSRI, other antidepressant, antipsychotic, benzodiazepine, and anticonvulsant users. Similar significant findings on overall DASS-21 scores were found for women at reproductive stage, with comorbidity of cPTSD, younger than 39 years old and those who used nomegestrol acetate/17-beta estradiol continuously during the study period.

**Table 4:** Adherence to nomegestrol acetate/17-beta estradiol and potential side effects for non-adherence at time 2 of interview.

Variables	n (%)
*Discontinued nomegestrol acetate/17-beta estradiol:* No Yes	n=49 31 (63.3%) 18 (36.7%)
*Reasons for discontinuing nomegestrol acetate/17-beta estradiol:*	*n=18*
Side effects	10 (55.6%)
Want pregnancy	1 (5.6%)
Did not help mood	4 (22.2%)
Didn’t need anymore	2 (11.1%)
Medical reasons	1 (5.6%)

**Table 5:** Paired-sample t-test for comparing pre-post overall DASS-21 scores (n=30)^a^.

DASS-21 scores	Mean (SD)	SE	T (*p*)	BCa 95%
***Clinic visit*** **Overall score**	27.27 (10.96)	2.00	4.72 (0.001)	6.46 - 14.26
***Phone call*** **Overall score**	17.03 (9.08)	1.65		

a Only women with scores from both clinic visit and phone call included.

## Discussion

In our pilot study, we evaluated the feasibility and clinical effectiveness of nomegestrol acetate/17-beta estradiol in women with PMDD. Over two-thirds of women adhered to nomegestrol acetate/17-beta estradiol during the study period, while around 20% of them reported discounting of the medication due to side effects. The number of women reporting a positive mood response to nomegestrol acetate/17-beta estradiol was much higher than for previously used COCPs and there was a significant reduction in their self-reported overall DASS-21 score.

The individual differences in response to a COCP could be due to the type of COCP and if the COCP was taken continuously or intermittently. The most common previously used COCP in our group was ethinylestradiol and levonorgestrel. Levonorgestrel is an older synthetic form of progesterone with androgenic properties and therefore may worsen mood symptoms in women with PMDD (34). The improved response to nomegestrol acetate/17-beta estradiol compared to other COCPs in our study could be due to both the different progesterone and estrogen in nomegestrol acetate/17-beta estradiol, a topic to be further explored in future studies.

Eighteen women in our study discontinued nomegestrol acetate/17-beta estradiol, with side effects being the most commonly provided reason. The rate of reported side effects from nomegestrol acetate/17-beta estradiol in our study appears higher than what has been previously reported by Mansour et al. (28) and Westhoff et al (29). In our study, breakthrough bleeding was the most common side effect in 40.8% of women compared to 11.7% and 9.1% reported in the two above studies, respectively. This discrepancy could be due to our small sample size but could also be due to our categorization of breakthrough bleeding. Of note, in the above studies, the incidence of breakthrough bleeding with nomegestrol acetate/17-beta estradiol was different to the number of women who identified breakthrough bleeding as a side effect. In both studies, approximately 30% of women had breakthrough bleeding in the first cycle and this decreased to approximately 12-25% across the remaining 12 cycles. We did not ask women when their breakthrough bleeding had occurred or how often it had occurred. It is known that breakthrough bleeding is common in women when initially taking a COCP. Nomegestrol acetate/17-beta estradiol is a low dose estrogen pill (estradiol alone is more rapidly metabolized than ethinylestradiol, which after liver metabolism persists as other estrogen metabolites), and this may be why women initially experience higher rates of breakthrough bleeding with nomegestrol acetate/17-beta estradiol compared with other COCPs. Nomegestrol has a longer half-life than other progestogens studied, which is consistent with its reduction in the frequency of bleeding days over time (28).

More women in our study reported weight gain (18.4%) compared to the 7.9% reported by Mansour et al (28) and the 9.5% reported by Westhoff et al (29). These two studies measured participants weight throughout the study period, whereas we asked for the women’s subjective opinion of weight changes since taking nomegestrol acetate/17-beta estradiol.

The secondary outcome in this study focused on clinical outcomes. This follow-up study suggests that nomegestrol acetate/17-beta estradiol may be an effective management option for mood symptoms in women with PMDD. The statistically significant improvements in the overall DASS-21 scores before and after treatment with nomegestrol acetate/17-beta estradiol, support our findings, that the majority of women reported a subjective positive mood response to nomegestrol acetate/17-beta estradiol.

Of note, there is a high prevalence of trauma in our population. 89.8% of women in our study had a history of trauma with cPTSD being a concurrent clinic diagnosis in 67.3% of women.

cPTSD occurs in individuals who have experienced prolonged traumatization and is characterised by emotional dysregulation, dissociation, somatization and poor self-esteem (35). It is understood that trauma and posttraumatic stress disorder (PTSD) are independently associated with PMDD (36). Our research suggests that stress can lead to numerous neurobiological changes, including neuroendocrine disruption of the hypothalamic-pituitary-adrenal axis (HPA) and hypothalamic-pituitary-gonadal axis (HPG). Feedback links between the HPA axis and the HPG axis may explain the connection between cPTSD and PMDD. Nomegestrol acetate/17-beta estradiol could be an effective adjunct treatment in women with PMDD and cPTSD to stabilize hormonal fluctuations that may contribute to emotional dysregulation.

Limitations of our study include our small sample size and the study design; in that it was an observational study with no comparison group. Given the retrospective nature of this study, there is a risk of recall bias in the women reporting their subjective mood response to nomegestrol acetate/17-beta estradiol and side effects, particularly more so for the women who were no longer taking nomegestrol acetate/17-beta estradiol. The DASS-21 score is not specific to PMDD and we did not specify the phase of the menstrual cycle women were at when they first completed the DASS-21 at the clinic, prior to commencing nomegestrol acetate/17-beta estradiol. We excluded women who had self-harming or suicidal behavior soon after taking nomegestrol acetate/17-beta estradiol for safety reasons which could have resulted in an overestimation of the positive effect of nomegestrol acetate/17-beta estradiol given that we excluded women who had a negative mood response. We also excluded women who used nomegestrol acetate/17-beta estradiol for less than one month, as it usually takes at least 1 month to see treatment effects. This may have introduced selection bias as there is a possibility the women who did not initially tolerate nomegestrol acetate/17-beta estradiol stopped taking the treatment after a few weeks. Furthermore, we did not take other factors into consideration that may influence mood and thus may change response to nomegestrol acetate/17-beta estradiol.

## Conclusions

This is the first study to investigate the feasibility and clinical effectiveness of nomegestrol acetate/17-beta estradiol treatment in the management of PMDD mood symptoms. Our findings support what we have seen in clinical practice and suggest that nomegestrol acetate/17-beta estradiol could be well tolerated by women suffering from PMDD and could be an effective first line treatment option for women with PMDD. Future research is required, particularly a randomized controlled trial of a large sample comparing nomegestrol acetate/17-beta estradiol to other recommended first line treatments for PMDD, either a drospirenone containing COCP or an SSRI.

## Author’s Note

The manuscript has been submitted solely to this journal and is not published in the press or submitted elsewhere.

## Data Availability Statement

The data analyzed in this study was obtained from routinely collected information as part of standard treatment in the Women's Mental Health Clinic, Alfred HREC project 285-17 ], the following restrictions apply: 1) That the aim/ purpose for the data access is clearly stated and must be for a genuine research purpose, 2) That the name and affiliation of the person/s requesting data access must be clearly stated 3) That the requesting individual/s must declare all and any conflict of interest - including past & current employment and any commercial involvement. 4) That any patient identifying details are kept confidentialRequests to access these datasets should be directed to the corresponding author: Professor Jayashri Kulkarni AM, jayashri.kulkarni@monash.edu.

## Ethics Statement

The studies involving human participants were reviewed and approved by The Alfred Ethics Committee. The patients/participants provided their written informed consent to participate in this study.

## Author Contributions

This clinical follow-up study was conceived by JK and CT. JK, consultant psychiatrist, had ultimate patient care responsibility. CT, endocrinologist, provided endocrine specialist care for patients. The initial questionnaire, phone calls, data collection, and data entry were completed by ER. NT assisted ER with the implementation of the objective DASS-21 scale and data analysis. In the revision and resubmission, LK, biostatistician, contributed enormously to the reanalyses and other work as per the reviewers’ suggestions. All authors contributed to the article and approved the submitted version.

## Conflict of Interest

The authors declare that the research was conducted in the absence of any commercial or financial relationships that could be construed as a potential conflict of interest.

## Publisher’s Note

All claims expressed in this article are solely those of the authors and do not necessarily represent those of their affiliated organizations, or those of the publisher, the editors and the reviewers. Any product that may be evaluated in this article, or claim that may be made by its manufacturer, is not guaranteed or endorsed by the publisher.
